# Factors associated with physical inactivity in adult breast cancer survivors—A population‐based study

**DOI:** 10.1002/cam4.1847

**Published:** 2018-10-24

**Authors:** Salam A. Huneidi, Nicole C. Wright, Arnisha Atkinson, Smita Bhatia, Purnima Singh

**Affiliations:** ^1^ Department of Epidemiology University of Alabama at Birmingham Birmingham Alabama; ^2^ Institute for Cancer Outcomes and Survivorship University of Alabama at Birmingham Birmingham Alabama

**Keywords:** age, breast cancer, pain, physical inactivity, survivorship, underweight

## Abstract

**Background:**

Physical activity has been shown to reduce the risk of breast cancer‐specific mortality. Although factors associated with physical inactivity in breast cancer survivors have been studied, a detailed examination at the population level is still lacking.

**Methods:**

We addressed this gap in 1236 women with a diagnosis of breast cancer from the 2016 Behavioral Risk Factor Surveillance System Cancer Survivorship module. Physical inactivity was defined as self‐reported absence of leisure time physical activity. Factors examined in the multivariable logistic regression model included sociodemographic, behavioral factors, access to health care, health history, current cancer treatment, and pain from cancer or treatment.

**Results:**

Overall, older age (≥65 years: OR = 2.63, 95% CI: 1.25‐5.55) and being underweight (BMI <18.5: OR = 6.11, 95% CI: 1.35‐27.66), were identified as significant factors associated with physical inactivity. In models adjusting for sociodemographics (Model 1), and the prior plus behavioral factors (Model 2), pain from cancer or treatment was significantly associated with physical inactivity (Model 2: OR = 2.23, 95% CI: 1.16‐4.28); however, after fully adjusting for all variables (Model 3), there was no longer evidence of a significant association between pain from cancer and physical activity in female survivors with breast cancer.

**Conclusions:**

We identified demographic (older age) and physical (low BMI and pain) factors to be significantly associated with physical inactivity among breast cancer survivors. Future interventions to promote physical activity in breast cancer survivors could benefit by taking into account these factors to develop tailored recommendations for increasing activity.

## 1 INTRODUCTION

Breast cancer is the most common cancer among women both in developed and developing countries.^1^ As of January 1, 2016, there were more than 3.5 million women in the United States with a history of breast cancer.^2^ Due to advances in early detection and treatment, there has been a growth in the number of cancer survivors, necessitating research to identify modifiable risk factors to prevent cancer recurrence and all‐cause mortality in survivors of breast cancer.^3^

Physical activity (PA) is associated with decreased cancer recurrence, increased survival and better health‐related quality of life in breast cancer survivors.^4^, ^5^, ^6^, ^7^, ^8^ In 2012, the American Cancer Society (ACS) created physical activity guidelines for cancer survivors that recommended at least 150 minutes of exercise per week.^9^ However, only an estimated 37% of breast cancer survivors adhere to the ACS exercise guidelines.^10^, ^11^, ^12^ As an example, data from the Behavioral Risk Factor Surveillance System (BRFSS) found that 31.5% of survivors had not engaged in any leisure time PA.^13^

PA is associated with weight loss and/or maintenance in healthy individuals^14^, ^15^ and recent studies have shown a beneficial effect of exercise on body weight regulation among breast cancer survivors.^16^, ^17^ Nearly half of the breast cancer survivors in the United States are ≥65 years of age,^18^ and the number of older breast cancer survivors is expected to increase in the coming years.^19^ Age at diagnosis has been found to be independently associated with an increased risk of breast cancer‐specific mortality,^20^ and there is a need to examine behaviors and outcomes among survivors of older age groups.^21^ For example, alcohol consumption increases estrogen levels and is linked to increased breast cancer risk and/or recurrence,^22^, ^23^, ^24^ but research has implied a positive relationship between physical activity and alcohol use.^25^, ^26^ Race could also be another factor associated with PA in breast cancer patients, as studies find that Black women are less likely to meet national PA guidelines compared to White cancer patients.^27^, ^28^, ^29^

Studies have found that while more than half of the cancer survivors were willing to engage in PA, poor health‐related factors (fatigue and joint stiffness), emotional and cognitive dysfunction, and environmental factors (lack of facilities and weather), were some of the factors that prevented them from engaging in PA.^30^ Specifically, cancer‐related factors such as pain, fatigue, financial hardship, and other sociodemographic factors serve as barriers to engaging in PA.^31^, ^32^ Pain is a common problem in cancer survivors and it is estimated that up to 50% of breast cancer survivors have chronic pain as a result of treatment with surgery, chemotherapy, and/or radiotherapy.^33^, ^34^, ^35^, ^36^, ^37^, ^38^ Patients with chronic pain avoid being active, which leads to deconditioning of the body and weight gain.^39^, ^40^ According to Brown et al,^41^ pain due to surgery or therapy impedes the recovery and rehabilitation of cancer survivors and negatively impacts their quality of life.

Few studies have evaluated barriers to PA in breast cancer survivors at the population level. The purpose of this cross‐sectional study was to examine factors associated with physical inactivity in breast cancer survivors by using data from the Cancer Survivorship Module in the 2016 BRFSS dataset.^42^ We focused on the following factors: sociodemographic, binge drinking, healthcare access, health history, and a separate model specifically focused on cancer‐related variables.

## 2 METHODS

### 2.1 Study population

The BRFSS is a population‐based telephone survey conducted annually in all 50 states, Washington, DC, and participating US territories to collect health information including health behaviors, preventive health practices, healthcare access, and chronic conditions among noninstitutionalized US adults ≥18 years of age.^43^ BRFSS is a “public‐use” de‐identified data set that does not require IRB approval for use in research. The median survey response rate in 2016 was 47%, and the median cooperation rate (the percentage of eligible persons contacted who completed the interview) was 70.5%.^42^ The 2016 BRFSS Cancer Survivorship module was administered in the following eight states: Idaho, Indiana, Louisiana, Michigan, Missouri, South Dakota, Virgin Islands, and Wisconsin.^42^ The data were weighted using poststratification methodology to adjust for the unequal probability of selection, differential nonresponse, and possible deficiencies in the sampling frame.^43^ As part of the BRFSS cancer survivorship module, respondents were asked about cancer type; 1236 women who indicated breast cancer diagnosis were included in the study. The BRFSS code book can be accessed from https://www.cdc.gov/brfss/annual_data/2016/pdf/codebook16_llcp.pdf.

### 2.2 Outcome variable: physical inactivity

Physical inactivity was based on a “no” response to the question “During the past month, other than your regular job, did you participate in any physical activities or exercises such as running, calisthenics, golf, gardening, or walking for exercise?” Those responding “yes” were considered physically active. Those with missing or invalid physical activity values were excluded (n = 4).

### 2.3 Independent variables

We examined the relationship between sociodemographic factors, binge drinking, healthcare access, health history, and cancer survivorship‐related variables, and physical inactivity. We chose these categories based on the previous physical activity studies in various cancer populations.^23^, ^24^, ^25^, ^26^, ^27^, ^28^, ^29^, ^30^, ^31^, ^32^, ^33^, ^34^, ^35^, ^36^, ^37^, ^38^, ^39^, ^40^, ^41^, ^42^ The following sociodemographic variables were included: age (18‐64, and ≥65 years), race/ethnicity (White, Black, and Other), education attainment (<high school and ≥high school), members in the household (1‐2, 3‐4 and >4), marital status (with partner vs without partner). We calculated the number of members in the household by adding the number of children and adults in a household (320 [25.6%] had missing data). For the marital status category, the “with partner” category included: “married” and “member of an unmarried couple,” and the “without partner” included “Divorced,” “Widowed,” “Separated,” and “Never married” survivors. The annual household income was categorized into <$25 000, $25 000 to <$50 000, ≥$50 000 (225 (18.1%) participants had missing income data), and the employment status was classified as employed, unemployed, unable to work, and other. Binge drinking was defined as women having ≥4 alcoholic drinks on one occasion in the past month.

Healthcare access variables included: healthcare coverage based on the “Do you have any kind of healthcare coverage?” with responses of “yes or no,” and “Could Not See Doctor Because of Cost” with responses of “yes or no.”.

We were interested in the following comorbidities: body mass index (BMI), heart attack/coronary heart disease (CHD) or myocardial infarction (MI), and diabetes. BMI was calculated as weight divided by the square of height (kg/m^2^ (underweight BMI <18.5 kg/m^2^, normal BMI = 18.5‐25 kg/m^2^), overweight (BMI = 25‐29.9 kg/m^2^), or obese (BMI ≥30 kg/m^2^) with 7.4% of the population have missing data (N = 92, Don't know/Refused/Missing). We were also interested in general health and physical and mental health status. General health was categorized as “excellent to good” and “fair to poor.” Survey respondents were asked how many days during the past 30 days they experienced poor physical and mental health, with responses in number of days.

Cancer‐related variables were based on the following questions: “Are you currently receiving treatment for cancer?” and “Do you currently have physical pain caused by your cancer or cancer treatment?” There was a moderate proportion of missing data (N = 285; 23%) for the “current pain” variable.

### 2.4 Statistical analysis

After preliminary review of the data for completeness and accuracy, we summarized the characteristics of the population by physical activity “yes” and “no” status using descriptive statistics that included frequencies, percentages or means, and standard deviations, depending upon each variable's scale of measurement and distribution. To assess the bivariate relationships, continuous and categorical variables were analyzed using independent sample t‐tests/Wilcoxon rank‐sum test and Fisher's exact/chi‐squared tests, respectively. All significant variables (*P* < 0.05) in the bivariate analysis and race were included in the multivariable logistic regression models to evaluate the association [odds ratios (OR) and 95% confidence intervals (CI)] with physical inactivity after accounting for survey design. Variables in Model 1 included the sociodemographic variables (Age, Race, Education, Marital Status, Income and Employment status). Model 2 included all variables in Model 1 and binge drinking, and finally, Model 3 included variables in Models 1, 2 and the comorbidity variables (BMI, Diabetes, CHD or MI, depressive disorder, general health and number of days when the physical and mental health was not good). Since pain from cancer or treatment can affect physical function significantly,^44^, ^45^, ^46^ we specifically evaluated the association between pain and physical inactivity by using separate multivariable logistic regression models. All analyses were performed in SAS, version 9.3, and all *P*‐values are 2‐sided and *P* < 0.05 was considered statistically significant.

## 3 RESULTS

The study sample included 1236 participants from the BRFSS 2016 cancer survivorship module dataset. A total of 376 (30.4%) breast cancer survivors were physically inactive. We compared the various factors of interest (sociodemographic, binge drinking, healthcare access, comorbidity, and pain) by physical inactivity status (Table 1). Physically inactive survivors were more likely to be older (≥65 years, *P* = 0.003), reported having ≤high school education (*P* < 0.0001), were without a partner (*P* < 0.0001), had lower annual household income (*P* < 0.0001) and were unable to work (*P* < 0.0001). Physically inactive survivors were over‐represented at both ends of the BMI spectrum; that is, were more likely to be underweight as well as obese (*P* = 0.005), were more likely to have diabetes (*P* < 0.0001), CHD or MI (*P* = 0.005) and depressive disorders (*P* = 0.0004). Physically inactive survivors were more likely to report fair/poor general health (*P* < 0.0001), reported a larger number of days when physical health or mental health was not good (*P* < 0.0001, *P* = 0.0004, respectively). Finally, physically inactive cancer survivors were more likely to report cancer‐related pain (*P* = 0.02).

**Table 1:** Characteristics of breast cancer survivors by physical activity status

Variables	Physical activity Yes (N = 860)	Physical activity No (N = 376)	Missing (N)	*P*‐value
Sociodemographics				
Age (N, %)			0	**0.0030**
18‐64	290 (47.56)	93 (33.79)		
≥65	570 (52.44)	283 (66.21)		
Race (N, %)			12	0.0670
White only, non‐Hispanic	769 (89.06)	312 (80.97)		
Black only, non‐Hispanic	55 (7.48)	43 (14.08)		
Other	30 (3.46)	15 (4.95)		
Education (N, %)			1	**<0.0001**
≤H.S	249 (34.28)	192 (56.73)		
>H.S	611 (65.72)	183 (43.27)		
Members in household			320	0.5550
1‐2	553 (77.46)	253 (81.31)		
3‐4	64 (18.32)	29 (13.25)		
>4	12 (4.22)	5 (5.45)		
Marital status (N, %)			9	**<0.0001**
Without partner	396 (33.37)	226 (52.06)		
With partner	457 (66.63)	148 (47.94)		
Income categories (N, %)			225	**<0.0001**
<25 000	169 (22.67)	138 (42.57)		
25 000 to <50 000	209 (26.81)	105 (31.59)		
≥50 000	326 (50.52)	65 (25.84)		
Employment status (N, %)			6	**<0.0001**
Employed	243 (32.73)	63 (20.01)		
Unable to work	38 (5.65)	55 (16.59)		
Unemployed	17 (2.71)	7 (1.60)		
Other	557 (58.90)	250 (61.80)		
Behaviors				
Binge drinking (N, %)			14	**0.0040**
Yes	58 (9.71)	14 (3.40)		
No	792 (90.29)	358 (96.60)		
Healthcare access				
Primary health insurance (N, %)			0	0.7700
Yes	848 (97.10)	366 (96.60)		
No	12 (2.95)	10 (3.45)		
Could not afford to see Doctor (N, %)			4	0.0700
Yes	37 (4.95)	27 (8.45)		
No	821 (95.05)	347 (91.55)		
Health history				
BMI (N, %)			92	**0.0050**
Underweight	14 (1.02)	5 (2.86)		
Normal	312 (36.96)	89 (26.01)		
Overweight	273 (35.66)	106 (32.75)		
Obese	198 (26.36)	147 (38.39)		
Diabetes (N, %)			35	**<0.0001**
Yes	125 (13.98)	101 (29.72)		
No	710 (86.02)	265 (70.28)		
CHD or MI (N, %)			18	**0.0050**
Yes	73 (6.89)	57 (14.27)		
No	777 (93.11)	311 (85.73)		
Depressive disorder (N, %)			2	**0.0004**
Yes	124 (17.22)	92 (30.60)		
No	735 (82.78)	283 (69.40)		
General health (N, %)			2	**<0.0001**
Excellent, very good, good	711 (82.45)	196 (52.08)		
Fair, poor	148 (17.55)	180 (47.92)		
Number of days physical health not good (mean, SD)	4.23 (8.57)	9.93 (12.14)	0	**<0.0001**
Number of days mental health not good (mean, SD)	2.60 (6.74)	4.20 (8.46)	0	**0.0004**
Cancer survivorship				
Current physical pain from cancer or treatment (N, %)			285	**0.0200**
Yes	88 (16.60)	45 (27.50)		
No		585 (83.40)	235	
Currently receiving treatment for cancer (N, %)			24	0.6700
Yes	137 (16.41)	65 (17.83)		
No	710 (83.59)	301 (82.17)		

Statistically significant *P*‐values are highlighted in bold.

Factors associated with physical inactivity among breast cancer survivors are presented in Table 2, including both crude and the various models after adjusting for sociodemographic factors (Model 1), sociodemographic factors and binge drinking (Model 2), and sociodemographic factors, binge drinking and comorbidities (Model 3) with their estimated odds ratios and 95% CI. After adjusting for the respective variables in the table and accounting for BRFSS survey weights, we found that those ≥65 years old had 2.6‐fold higher odds of being physically inactive compared to 18‐64 year‐olds (OR = 2.63 95% CI: 1.25‐5.55), and underweight survivors had 6.1‐fold higher odds of being physically inactive compared to normal weight survivors (OR = 6.11 95% CI: 1.35‐27.66) (Table 2). This association was positive in all the three models. Additionally, we found that lower education (≤HS) was associated with twofold greater odds and inability to work with threefold greater odds of physical inactivity in both Models 1 and 2. However, there was no evidence of these factors being associated with physical inactivity when adjusted for comorbidities in Model 3. There was a marginal association between physical inactivity and “days not feeling good” (OR = 1.04 95% CI: 1.00, 1.08).

**Table 2:** Factors associated with physical inactivity in breast cancer survivors

	Crude OR (95% CI)	Adjusted OR (95% CI)[Link]	Adjusted OR (95% CI)[Link]	Adjusted OR (95% CI)[Link]
		Model 1	Model 2	Model 3
Sociodemographics				
Age				
18‐64	Ref	Ref	Ref	Ref
≥65	**1.78 (1.21, 2.61)**	**3.13 (1.71, 5.71)**	**2.97 (1.60, 5.50)**	**2.63 (1.25, 5.55)**
Race				
White	Ref	Ref	Ref	Ref
Black	**2.07 (1.11, 3.86)**	1.37 (0.63, 2.95)	1.35 (0.63, 2.91)	1.47 (0.68, 3.19)
Other	1.57 (0.55, 4.52)	1.83 (0.48, 6.93)	1.72 (0.45, 6.63)	1.21 (0.20, 7.21)
Education				
>H.S	Ref	Ref	Ref	Ref
≤H.S	**2.51 (1.75, 3.61)**	**1.99 (1.20, 3.29)**	**1.92 (1.17, 3.17)**	1.48 (0.82, 2.69)
Marital status				
With partner	Ref	Ref	Ref	Ref
Without partner	**2.17 (1.52, 3.09)**	1.12 (0.68, 1.85)	1.09 (0.66, 1.80)	1.11 (0.62, 1.98)
Income categories				
≥50 000	Ref	Ref	Ref	Ref
25 000 to <50 000	**2.30 (1.43, 3.72)**	1.22 (0.66,2.23)	1.17 (0.63, 2.15)	1.22 (0.64, 2.31)
<25 000	**3.67 (2.26, 5.96)**	1.74 (0.88,3.44)	1.78 (0.89, 3.55)	1.95 (0.87, 4.38)
Employment status				
Employed	Ref	Ref	Ref	Ref
Unable to work	**4.80 (2.24, 10.26)**	**2.97 (1.18,7.44)**	**2.82 (1.13, 7.02)**	1.47 (0.45, 4.83)
Unemployed	0.97 (0.29, 3.25)	0.77 (0.14, 4.32)	0.80 (0.14, 4.66)	0.54 (0.06, 4.55)
Other	**1.72 (1.06, 2.79)**	0.85 (0.45, 1.59)	0.86 (0.46, 1.64)	0.88 (0.39, 1.97)
Behaviors				
Binge drinking				
No	Ref		Ref	Ref
Yes	**0.33 (0.15, 0.73)**		0.45 (0.15, 1.36)	0.61 (0.19, 2.02)
Health history				
BMI				
Normal	Ref			Ref
Underweight	**3.99 (1.01, 15.75)**			**6.11 (1.35, 27.66)**
Overweight	1.31 (0.82, 2.09)			1.58 (0.79, 3.16)
Obese	**2.07 (1.33, 3.23)**			1.55 (0.83, 2.87)
Diabetes				
No	Ref			Ref
Yes	**2.60 (1.66, 4.07)**			1.42 (0.73, 2.73)
CHD or MI				
No	Ref			Ref
Yes	**2.25 (1.26, 4.02)**			1.53 (0.66, 3.55)
Depressive disorder				
No	Ref			Ref
Yes	**2.12 (1.40, 3.22)**			1.47 (0.74, 2.90)
General health				
Excellent, very good, good	Ref			Ref
Fair, poor	**4.32 (2.86, 6.53)**			1.86 (0.93, 3.73)
Number of days physical health not good	**1.06 (1.04, 1.08)**			1.04 (1.00, 1.08)
Number of days mental health not good	**1.03 (1.01, 1.06)**			0.99 (0.96, 1.03)

Model 1: Adjusted for sociodemographic factors.

Model 2: Adjusted for Model 1 + behaviors.

Model 3: Adjusted for Model 1, 2 + health history.

CI, confidence interval; OR, odds ratio.

Estimated using logistic regression. Statistically significant OR and CI are highlighted in bold.

To assess the relationship between physical inactivity and cancer‐related pain in detail, we again conducted a series of analyses adjusting for sociodemographic factors (Model 1), sociodemographic factors and binge drinking (Model 2), and sociodemographic, binge drinking and comorbidities (Model 3) (Table 3). Cancer‐related pain was significantly associated with physical inactivity in Model 1 (OR = 2.17, 95% CI: 1.13, 4.17) and Model 2 (OR = 2.23, 95% CI: 1.16‐4.28) but there was no evidence of an association after adjusting for the variables in Model 3 (OR = 1.87, 95% CI: 0.77‐4.53).

**Table 3:** The association between pain from cancer or treatment and physical activity

Current pain from cancer or treatment	Crude OR (95% CI)	Model 1 OR (95% CI)[Link]	Model 2 OR (95% CI)[Link]	Model 3 OR (95% CI)[Link]
Yes vs no	**1.91 (1.10, 3.32)**	**2.17 (1.13, 4.17)**	**2.23 (1.16, 4.28)**	1.87 (0.77, 4.53)

Model 1: Adjusted for sociodemographic factors.

Model 2: Adjusted for Model 1 + behaviors.

Model 3: Adjusted for Model 1, 2 + health history.

CI, confidence interval; OR, odds ratio.

Estimated using logistic regression. Statistically significant OR and CI are highlighted in bold.

## 4 DISCUSSION

Physical activity is recommended for cancer survivors to enhance their health and quality of life. Regular PA can increase recurrence‐free survival rates of cancer patients.^6^, ^7^, ^8^, ^9^, ^10^ About two‐thirds of cancer survivors do not adhere to ACS exercise guidelines due to multiple sociodemographic, economic, health, and cancer‐related factors that could prevent them from regularly engaging in physical activities.^11^ In the present study, we examined the relationship between physical and psychosocial factors associated with physical inactivity in adult breast cancer survivors. Overall, we found that older age and being underweight are significant risk factors for physical inactivity in breast cancer survivors.

Our findings are consistent with previously published findings by Kampshoff et al^31^ who utilized data from 574 female breast cancer survivors from three different lifestyle intervention studies in Australia and New Zealand, who reported older age as a significant barrier to physical activity. Kampshoff et al^31^ also found that higher BMI and presence of comorbidities were associated with physical inactivity. We found associations between BMI and the comorbidities assessed in our crude analyses, but these factors did not remain significant after adjustment. Similar to our study, Kampshoff et al^31^ did not show a significant association of PA with marital status and all treatment‐related characteristics.

Although we did not find an association with high BMI, we did observe a negative effect of being underweight on physical activity. However, there were only nineteen underweight individuals and of these only five were physically inactive in our dataset. It has been reported that women who are underweight before breast cancer diagnosis are at the greatest risk of all‐cause mortality,^47^, ^48^ possibly because being underweight could be a sign of poor general health or malnutrition which may affect the woman's ability to exercise due to poor muscle mass index. In the current study, we could not explore whether malnutrition was an issue as no nutrition data were available in the BRFSS dataset.

Brunet et al^49^ found that fatigue, pain and a lack of energy were major factors that breast cancer survivors perceived as barriers to performing physical activity. Likewise, Blaney et al^30^in their cross‐sectional study also reported that pain and fatigue were among the top 10 factors that interfered with participation in physical activity. Although not significant after adjusting for all sociodemographic, behavioral, and health history factors, we found that cancer pain was associated with twofold higher odds of being physically inactive in Models 1 and 2, when adjusted for sociodemographic and binge drinking.

We did not find race/ethnicity to be a significantly associated with physical inactivity. This could potentially be attributed to small sample sizes for Black and Other race categories. Previous studies in which Black or other minority samples were sufficiently large, a significant association between race and physical activity was reported. Lu et al^50^ also reported racial difference in physical activity in breast cancer survivors; Asian American women reported the lowest level of recreational physical activity, followed by Latinas. Hair et al^27^ also found that Black women were less likely to meet national physical activity guidelines and reported lower levels of pre‐diagnosis and post‐diagnosis physical activity.

The findings in this report are subject to several limitations. First, BRFSS is a random‐digit–dialed telephone survey where the information is reported directly by the respondent, so it may be subject to information based on social desirability, which could lead to inaccurate estimates of physical inactivity as well as information bias around the factors associated with PA. As BRFSS is a survey of community dwelling adults, it would not capture women with breast cancer who are in nursing homes and/or hospice care, whose physical activity may be limited based on their disease. The “Cancer Survivorship” module was included in BRFSS only in 2016 and is limited to thirteen questions. The “cancer” specific questions do not include disease‐related variables such as cancer stage and type of cancer treatment. Also, years since diagnosis is not reported and could not be calculated, as exact “current age” is not recorded but categorized as 1‐24, 25‐34 etc years. Smoking behavior could not be included in the analysis due to significant missing values. In addition, the underweight category for the BMI variable had a small sample size and can affect the generalizability of the results. The results are from a cross‐sectional study, and due to the fact that both risk factors and outcome(s) are measured simultaneously, causal inferences cannot be determined.

However, there are strengths to our study. To our knowledge, this is one of the few studies to evaluate factors associated with physical inactivity in breast cancer survivors by sociodemographic factors, psychosocial factors, comorbidities, and pain from treatment or cancer at the population level. We have also used the most recent BRFSS dataset and cancer survivorship module to evaluate the barriers to physical activity in adult breast cancer survivors.

## 5 CONCLUSIONS

In our population‐based study, age (older) and BMI (underweight) are significant risk factors for physical inactivity among adult breast cancer survivors. Additionally, pain from cancer or treatment was significantly associated with physical inactivity. Providers should routinely screen patients for physical inactivity, provide recommendations for increasing activity and implement appropriate lifestyle interventions that could help breast cancer survivors adopt and maintain a healthy behavior to potentially reduce morbidity and mortality. Additional research is needed to understand why older and underweight survivors are at highest risk of inactivity so that appropriate tailored interventions can be developed.

## CONFLICT OF INTEREST

The authors declare that there is no conflict of interest regarding the publication of this article. NCW has received research funding from Amgen unrelated to this project, and is a consultant for Pfizer, Canada.

## AUTHOR CONTRIBUTIONS

Salam A. Huneidi involved in the data curation, formal analysis, methodology, writing of the review and editing. Nicole C. Wright involved in the methodology and writing of the review and editing. Arnisha Atkinson involved in the data curation and writing the original draft. Smita Bhatia involved in the writing of the review and editing. Purnima Singh involved in the conceptualization, supervision, data curation, methodology, formal analysis, writing the original draft, and writing of the review and editing.
